# Towards globally equitable bioinformatics adoption

**DOI:** 10.1371/journal.pbio.3003839

**Published:** 2026-07-01

**Authors:** Paulyna Magaña, Piraveen Gopalasingam, Jennifer R. Fleming, Oleg Kovalevskiy, Augustin Žídek, ThankGod Echezona Ebenezer, Agata Laydon, Roz Onions, Eva Akurut, Syed Muktadir Al Sium, Yalbi Itzel Balderas-Martínez, Sanjana Fatema Chowdhury, Saikat Chowdhury, Sylvia Christie, Govinda Rao Dabburu, Fatoumata Gnine Fofana, Ronald Galiwango, Mahipal Ganji, Daudi Jjingo, Fredrick Elishama Kakembo, Grace Kebirungi, Shahiid Kiyaga, Ayoub Ksouri, Sanjeet Kumar Mahtha, Vinicius Maracaja-Coutinho, Jack Mason, Jose Arturo Molina-Mora, Patricia P. N. Nabisubi, Emmanuel Nji, Houcemeddine Othman, Martina Soledad Paoletta, Nicole M. Scherer, Bhagya Senadheera, Adrián Gustavo Turjanski, David Twesigomwe, Andrew Walakira, Sameer Velankar, Cath Brooksbank

**Affiliations:** 1 EMBL-European Bioinformatics Institute, Wellcome Genome Campus, Hinxton, Cambridge, United Kingdom; 2 Google DeepMind, London, United Kingdom; 3 African Center of Excellence in Bioinformatics and Data-Intensive Science, Infectious Diseases Institute, College of Health Sciences, Makerere University, Kampala, Uganda; 4 Bangladesh Council of Scientific and Industrial Research, Dhaka, Bangladesh; 5 Instituto Nacional de Enfermedades Respiratorias Ismael Cosio Villegas, Mexico City, Mexico; 6 CSIR-Centre for Cellular and Molecular Biology, Hyderabad, Telangana, India; 7 Academy of Scientific and Innovative Research, Ghaziabad, Uttar Pradesh, India; 8 University of Delhi, South Campus, New Delhi, India; 9 African Centre of Excellence in Bioinformatics and Data Sciences (ACE-Mali), University of Sciences, Techniques and Technologies of Bamako (USTTB), Bamako, Mali; 10 Department of Biochemistry, Indian Institute of Science, Bangalore, India; 11 Department of Immunology and Molecular Biology, Makerere University College of Health Sciences, Kampala, Uganda; 12 Infectious Diseases Research Collaboration (IDRC), Kampala, Uganda; 13 Sydney Brenner Institute for Molecular Bioscience, Faculty of Health Sciences, University of the Witwatersrand, Johannesburg, South Africa; 14 National Institute of Immunology, New Delhi, India; 15 Unidad de Genómica Avanzada (UGA), Advanced Center for Chronic Diseases (ACCDiS), Facultad de Ciencias Químicas y Farmacéuticas, Universidad de Chile, Santiago, Chile; 16 Laboratório de Medicina e Saúde Pública de Precisão (MESP2), Instituto Gonçalo Moniz, Fundação Oswaldo Cruz, Fiocruz, Salvador, Brazil; 17 Instituto Nacional de Ciência e Tecnologia em Saúde Digital (INCT-DigiSaúde), Salvador, Brazil; 18 Centro de Investigación en Enfermedades Tropicales, Centro de Investigación en Hematología y Trastornos Afines, and Facultad de Microbiología, Universidad de Costa Rica, San José, Costa Rica; 19 BioStruct-Africa, Stockholm, Sweden; 20 BioStruct-Africa, Kumasi, Ghana; 21 BioStruct-Africa, Nairobi, Kenya; 22 Department of Parasitology and Microbiology, Centre for Research in Infectious Diseases, Yaounde, Cameroon; 23 Membrane Protein Laboratory, Diamond Light Source, Research Complex at Harwell, Didcot, United Kingdom; 24 Department of Genetics, Farhat Hached University Hospital, Sousse, Tunisia; 25 Laboratory of Cytogenetics, Molecular Genetics and Reproductive Biology (LR03SP02), Farhat Hached University Hospital, Sousse, Tunisia; 26 Instituto de Agrobiotecnología y Biología Molecular (IABIMO), INTA-CONICET, Hurlingham, Argentina; 27 Bioinformatics Core, Brazilian National Cancer Institute (INCA), Rio de Janeiro, Brazil; 28 The Allergy, Immunology and Cell Biology Unit, Faculty of Medical Sciences, Colombo, Sri Lanka; 29 Universidad de Buenos Aires, Buenos Aires, Argentina; 30 IQUIBICEN-CONICET, Buenos Aires, Argentina; 31 Medical Research Council/Uganda Virus Research Institute and London School of Hygiene and Tropical Medicine Uganda Research Unit, Entebbe, Uganda

## Abstract

Advances in AI-driven bioinformatics promise democratized discovery, yet major inequities persist. This Perspective uses AlphaFold as an illustrative case to argue that equitable adoption of bioinformatics technologies will require sustained global investment, not just provision of access.

## Teaser

Advances in artificial intelligence (AI)-driven bioinformatics promise democratized discovery, yet major inequities persist. Equitable adoption of bioinformatics tools will require sustained investment in infrastructure, training, institutions, and global communities, not just access.

Advances in bioinformatics and artificial intelligence (AI) have reshaped the life sciences, enabling large-scale data analysis, predictive modeling, and integrative approaches across disciplines [1,2]. Many contemporary bioinformatics tools and AI models are released as open-source resources, with publicly accessible code repositories, pre-trained models, and web-based interfaces. This widespread availability has reinforced the expectation that technical barriers to participation have largely been removed; however, experience across multiple global locations reveals a persistent disconnect between open access and open utility. Analyses conducted by Google DeepMind on AlphaFold citation data using OpenAlex [3] and national diagnostics show that researchers in low- and middle-income countries (LMICs) remain underrepresented in the development and downstream application of advanced bioinformatics tools, even when these tools are freely available. These patterns are consistent with long-standing disparities in computational infrastructure, training capacity, and institutional investment [4,5]. Ensuring equitable adoption of bioinformatics tools will therefore require more than making the tools themselves open access (Box 1).

**Box 1. Priorities for equitable adoption of advanced bioinformatics tools.**

**1. Enable shared infrastructure and low-barrier computational access.**

For example: foster collaborations with cloud computing providers, form partnerships with high-performance computing centers or well-resourced institutions, and develop training materials tailored for offline use and low-bandwidth environments.

**2. Invest in scalable Train-the-Trainer and mentorship models.**

For example: create training resources suitable for various delivery methods, such as in-person, online, and hybrid formats, and make them openly available with guidance on how to use them.

**3. Prioritize language accessibility and contextual relevance.**

For example: translate training materials into the local language and provide use cases relevant to areas of direct interest to the trainees.

**4. Integrate bioinformatics training into institutional curricula and funding structures.**

For example: incorporate training on bioinformatics tools into undergraduate and taught master’s curricula; provide early-career researchers with access to short courses.

**5. Foster global, interdisciplinary communities of practice.**

For example: organize regional hubs or collaborative clusters to address geopolitical and logistical challenges and foster collaboration both inter-country and among neighboring countries.

The advent of AI has profoundly transformed bioinformatics applications, transitioning many from rigid, deterministic local pipelines to scalable, data-driven computations that demand far greater computational resources. AlphaFold 2 exemplifies this broader structural challenge. Its release, together with the AlphaFold Protein Structure Database, made large-scale predicted structures broadly accessible in principle [1,6], yet routine use and methodological innovation remain concentrated in settings with established computational infrastructure and specialist expertise [4,7,8]. Similar patterns have long been documented across genomics, transcriptomics, metagenomics, and public health bioinformatics, indicating that these challenges are systemic and long-standing rather than specific to any single tool or technological generation [9–11].

The arguments presented here draw on both published analyses and structured discussions held during the AlphaFold Education Summit, which brought together educators and researchers from multiple regions, with strong representation from LMICs (see the full report here). Across both literature and summit discussions, access to computational infrastructure was described as a central limitation. Many contemporary bioinformatics methods depend on high-performance computing (HPC), graphic processing units (GPU), large-scale storage, and reliable internet connectivity, yet in many LMIC contexts, computational resources are decentralized and siloed within selected institutions, limiting broader accessibility [5,11]. These limitations are further compounded by unreliable power supplies and low-bandwidth internet connectivity. Under these conditions, cloud-based solutions, despite occasionally being designed to minimize reliance on computing infrastructure (e.g., the AlphaFold Database) and promoted as accessible tools, may remain difficult to use reliably and sustainably, particularly given their ongoing cost implications. In these contexts, the mere existence of sophisticated software, and even access to it, provides limited practical benefit.

Training limitations further compound these constraints. Regional capacity assessments and mentorship program evaluations consistently report shortages of trained bioinformaticians, instructors, and infrastructure administrators in resource-limited settings [4,5]. Training initiatives are frequently short-term and externally driven, providing limited opportunities for progression or local ownership. Evidence from mentorship-based programs and long-standing regional networks indicates that one-off workshops rarely yield sustained expertise, whereas longer term, mentored approaches are effective in building local capacity to sustain these efforts, thereby fostering independent analytical capability and local leadership [4,10].

Language and contextual accessibility further influence participation, and are often under-recognized barriers. Evaluations of training initiatives in LMICs emphasize the importance of locally relevant datasets, multilingual materials, and adaptive adult-aimed design [12]. Summit participants echoed these findings, highlighting that sustained engagement depends not only on technical instruction, but on researchers’ ability to apply tools independently within locally relevant research contexts. Furthermore, securing adequate and sustained funding for training programs, essential infrastructure, and long-term community-building initiatives remains a significant concern, as it threatens continuity and contributes to cycles of capacity loss.

Drawing on both summit deliberations and established regional initiatives, five interrelated priorities emerge for equitable adoption of advanced bioinformatics tools (Box 1). Central among these is resource-aware design: developing training materials suitable for low-bandwidth environments, including pre-configured notebooks and locally deployable workflows, and fostering collaborations with HPC centers and cloud providers to mitigate computational constraints. Ensuring that host institutions possess baseline hardware capacity is equally important.

Another critical element is scalable training and mentorship networks. A core feature of the AlphaFold Education Summit was a Train-the-Trainer model [13], designed to strengthen local training capacity by integrating technical instruction with andragogical development and community-building strategies. This approach aligns with earlier regional initiatives such as BioStruct-Africa [8], CABANAnet in Latin America [14], and APBioNet in the Asia–Pacific region [9], which demonstrate that capacity building is most effective when organized around communities of practice rather than individual platforms. Such networks enable peer mentorship, shared infrastructure, and engagement with science policy across evolving technological landscapes [10,14]. Such models, when embedded within longer term mentorship frameworks, support skill retention, independence, and the development of local research leadership [4,7,10]. These approaches move beyond episodic instruction toward sustained institutional capacity.

Language accessibility and contextual relevance are prerequisites for inclusion. The careful development of multilingual training materials, validated by local experts, and the grounding of instruction in locally relevant research questions, strengthen learner confidence and facilitate integration of new tools into existing research programs. Finally, institutional and policy integration are essential for sustainability. Embedding bioinformatics training within university curricula, aligning initiatives with national research strategies, and supporting stable career pathways are paramount to retaining expertise and avoiding cycles of capacity loss [8]. Collaborative funding models, including institutional consortia, industry partnerships, and philanthropic engagement, may further enhance resilience.

The rapid expansion of AI-enabled and resource-intensive bioinformatics tools makes it clear that democratization cannot be assumed to follow from open access alone. AlphaFold illustrates how even widely celebrated open access tools can reproduce existing inequities when infrastructure, training, and institutional support remain unevenly distributed. Ensuring equitable participation, therefore, requires deliberate, sustained investment in shared infrastructure, long-term training networks, language accessibility, institutional integration, and global communities of practice. Without such commitments, the benefits of technological innovation risk remaining concentrated in already well-resourced settings. Treating equity as integral to scientific infrastructure, embedded within funding decisions, institutional planning, and capacity building policy, offers a path toward a more inclusive and globally representative research ecosystem.

## Abbreviations

AI: artificial intelligence
HPC: high-performance computing
IDRC: Infectious Diseases Research Collaboration
LMICs: low- and middle-income countries.
